# An Insulator Element Located at the Cyclin B1 Interacting Protein 1 Gene Locus Is Highly Conserved among Mammalian Species

**DOI:** 10.1371/journal.pone.0131204

**Published:** 2015-06-25

**Authors:** Wataru Yoshida, Junko Tomikawa, Makoto Inaki, Hiroshi Kimura, Masafumi Onodera, Kenichiro Hata, Kazuhiko Nakabayashi

**Affiliations:** 1 Department of Maternal-Fetal Biology, National Research Institute for Child Health and Development, Setagaya, Tokyo, Japan; 2 Department of Human Genetics, National Research Institute for Child Health and Development, Setagaya, Tokyo, Japan; 3 Department of Biological Sciences, Graduate School of Bioscience and Biotechnology, Tokyo Institute of Technology, Yokohama, Japan; Duke University, UNITED STATES

## Abstract

Insulators are *cis*-elements that control the direction of enhancer and silencer activities (enhancer-blocking) and protect genes from silencing by heterochromatinization (barrier activity). Understanding insulators is critical to elucidate gene regulatory mechanisms at chromosomal domain levels. Here, we focused on a genomic region upstream of the mouse *Ccnb1ip1* (cyclin B1 interacting protein 1) gene that was methylated in E9.5 embryos of the C57BL/6 strain, but unmethylated in those of the 129X1/SvJ and JF1/Ms strains. We hypothesized the existence of an insulator-type element that prevents the spread of DNA methylation within the 1.8 kbp segment, and actually identified a 242-bp and a 185-bp fragments that were located adjacent to each other and showed insulator and enhancer activities, respectively, in reporter assays. We designated these genomic regions as the *Ccnb1ip1* insulator and the *Ccnb1ip1* enhancer. The *Ccnb1ip1* insulator showed enhancer-blocking activity in the luciferase assays and barrier activity in the colony formation assays. Further examination of the *Ccnb1ip1* locus in other mammalian species revealed that the insulator and enhancer are highly conserved among a wide variety of species, and are located immediately upstream of the transcriptional start site of *Ccnb1ip1*. These newly identified cis-elements may be involved in transcriptional regulation of *Ccnb1ip1*, which is important in meiotic crossing-over and G2/M transition of the mitotic cell cycle.

## Introduction

Insulators are DNA cis-elements that serve as a boundary element to organize chromatin domains. Insulators have two functions: the enhancer-blocking activity that prevents promoter-enhancer interaction, and the barrier activity that prevents the spread of heterochromatin. The first identified insulators were specialized chromosome structures (scs) and scs’ that are located in the vicinity of the Drosophila hsp70 gene [1–3]. Thereafter, insulators have been identified in several species such as yeast, sea urchins, chickens, mice and humans [4].

In vertebrates, the most-characterized insulator is the cHS4 insulator located in the chicken β-globin locus [5]. The cHS4 insulator was identified as a 1.2 kbp DNaseI-hypersensitive region. The hypersensitive region contains a 250 bp core sequence that shows insulator activity [6]. CTCF, which is required for enhancer-blocking activity, binds within a 42 bp region in the core sequence [7]. USF1/USF2 and VEZF1 have been shown to bind to the other region of the core sequence and to mediate barrier activity [8–10]. The barrier and the enhancer-blocking activities of the cHS4 insulator have been utilized to prevent transgene silencing by position effect [11–13] and to suppress transgene-mediated insertional transcriptional activation [14,15], respectively. Identification and characterization of novel insulators are expected to contribute to the further development of effective vectors for gene therapy and transgenesis [16]. Mutations at endogenous insulators disrupt normal gene regulation, and could cause diseases. Such insulator mutations have been reported for facioscapulohumeral muscular dystrophy (the D4Z4 insulator) and hereditary spherocytosis (the ankyrin-1 gene insulator) [17,18]. Therefore, identification of insulators could also contribute to elucidate endogenous gene regulation and disease mechanisms.

The mouse *Parp2* and *Ccnb1ip1* genes encode ADP-ribosyltransferase 2 and cyclin B1 interacting protein 1, respectively, and are located next to each other in a head-to-head orientation intervened with a 12 kb intergenic region. In addition to its well-known role in DNA repair, *Parp2* is shown to have essential functions in both meiosis I and spermiogenesis [19]. The protein encoded by *Ccnb1ip1* is a putative ubiquitin E3 ligase, disruption of which is shown to result in meiotic defects in both sexes [20]. The human CCNB1IP1 protein was originally identified as the human enhancer of invasion clone 10 (HEI10), and demonstrated to be a RING-finger family ubiquitin ligase that regulates cell cycle progression through G2/M by interacting with cyclin B and promoting its degradation [21]. CCNB1IP1 (HEI10) has been shown to interact with the tumor suppressor protein Merlin (encoded by the neurofibromatosis 2 gene) [22], and to negatively regulate cell invasion by inhibiting cyclin B/Cdk1 and other promotility proteins [23]. A large-scale *in-situ* hybridization study on cancerous tissues showed that *CCNB1IP1* was underexpressed in breast cancer and most likely non-small cell lung cancer [24]. CCNB1IP1 may be commonly involved in coordinating cell cycles with cell migration and invasion. Therefore, understanding epigenetic mechanisms that control the transcription of *Parp2* and *Ccnb1ip1* helps gain further insights into their functional roles in meiosis, mitosis, and tumor development.

In the course of examining the DNA methylation status of the genomic interval between the *Parp2* and the *Ccnb1ip1* genes, we identified a region methylated in the day 9.5 embryo of the C57BL/6 (B6) strain but not methylated in that of the JF1/Ms strain at the upstream region of the *Ccnb1ip1* gene. By further characterizing this locus, we identified a 1.8 kbp genomic segment present in the 129X1/SvJ and JF1 strains but not in the B6 strain, and revealed that the 1.8 kbp genomic segment contains a boundary between methylated and unmethylated regions. Several insulators have been reported to prevent DNA methylation [10,12,25–29]; therefore, we investigated whether the 1.8 kbp segment contains an insulator-type element that may be involved in the establishment of the identified DNA methylation boundary.

## Materials and Methods

### Mice

This study was carried out in strict accordance with the recommendations in the Guide for the Care and Use of Laboratory Animals of the National Research Institute for Child Health and Development (NCCHD) of Japan. The protocol was approved by the Committee on the Ethics of Animal Experiments of NCCHD (Permit Number: 2010–002). Two inbred strains, C57BL/6 and JF1/Ms (Japanese Fancy Mouse), were used. Pregnant female mice at embryonic day 9.5 (E9.5) were sacrificed by cervical dislocation, and all efforts were made to minimize suffering. E9.5 embryos were used for bisulfite methylation, RT-PCR, Chromatin immunoprecipitation analyses.

### Bisulfite methylation analysis

Bisulfite treatment of genomic DNAs was performed by EpiTect Bisulfite Kit (QIAGEN). Bisulfite-PCR was performed using EX Taq HS (Takara). The PCR products were cloned using the StrataClone PCR cloning kit (Agilent). Individual clones were sequenced using ABI PRISM 3130xl DNA Sequencer. PCR primers (S1 Table) were designed using the MethPrimer web server [30].

### Cell culture

Cell lines were cultured under standard culture conditions (at 37°C, 5% CO_2_ in air) in DMEM medium (Sigma D6429) supplemented with 10% fetal bovine serum and Penicillin-Streptomycin-Glutamine Liquid (final 1x, GIBCO 10378–016).

### Reporter assay

The target genomic regions were amplified by PCR. Insulators and enhancers were connected by overlap PCR. The PCR products were cloned in the *Sfi*I site of the pGL4.23 [luc2/minP] (Promega) and then transformed into *E*. *coli* JM110 (Stratagene). The plasmids were purified using Wizard Plus SV Minipreps DNA Purification System (Promega). Cos7, NIH3T3 or HeLa cells were transfected with a reporter vector (0.4 pmol) and Renilla control vector (0.2 ng) by Lipofectamine 2000 (Invitrogen). After 48h, cells were harvested and measured for luciferase activities using the Dual-Luciferase Reporter Assay System (Promega). Primers (S2 Table) were designed using the Primer3 web server [31]. Luciferase reporter assays were performed in triplicates.

### Colony assay

The neomycin-resistant gene was cloned into pTA2 vector (Toyobo). Subsequently, the *Ccnb1ip1* or cHS4 insulator was cloned into both of *Xba*I-*Sac*II and *Xho*I-*Cla*I sites (primers used are listed in S3 Table). Cos7 cells were transfected with a vector by Lipofectamine 2000 (Invitrogen). After 24h, 4000 cells each were seeded to two 100mm dishes: one was cultured with G418 (800 μg/ml) and the other without G418. After 13 days, resultant colonies were stained with giemsa’s azur eosin methylene blue solution (Merck) and counted. Colonies assays were performed in triplicates.

### Reverse transcription (RT)-PCR

Total RNAs from E9.5 embryos were isolated using AllPrep DNA/RNA Mini kit (QIAGEN). cDNA was synthesized from 1 μg of total RNA using QuantiTect Reverse Transcription kit (QIAGEN). Quantitative RT-PCR was carried out using 1/100 of the resultant cDNA as template DNA and the SYBR Premix Ex Taq (Takara) in a 25 μl reaction volume on the 7900HT Fast Real-Time PCR System (Applied Biosystems) under the following PCR cycle conditions: 95°C for 5 min, 40 cycles of [95°C for 30 s, 59°C for 30 s, 72°C for 30 s]. PCR primers used are listed in S4 Table. The expression levels of *Ccnb1ip1* and *Parp2* were normalized by that of *Gapdh*. For the sequencing of RT-PCR products, PCR was performed using EX Taq HS (Takara) (primers listed in S4 Table). The PCR products were treated with ExoSAP-IT (GE Healthcare) and subjected to direct sequencing using the PCR primers as sequencing primers.

### Chromatin immunoprecipitation (ChIP) assays and quantitative PCR for histone modifications

ChIP assays for histone H3K4me3, H3K9me3, H3K27me3 were performed as described previously [32] with modifications. Four each of E9.5 embryos of B6 and JF1 strains were pooled, fixed with 1% formaldehyde for 5 min, resuspended in SDS lysis buffer (ChIP Reagent #318–07131, Nippon Gene), and sonicated using a S220 Focused-ultrasonicator (Covaris). The antibodies used were anti-H3K4me3 antibody (CMA304/16H10) [33], anti-H3K9me3 antibody (CMA318/2F3) [34], and anti-H3K27me3 (CMA323/1E7) [34]. Normal mouse IgG (Millipore, 12–371) was used as a control. Sonicated chromatin equivalent to approximately 5 x 10^5^ cells was added with ChIP dilution buffer (ChIP Reagent #318–07131, Nippon Gene) and 2 μg of an antibody (or control IgG) pre-coated onto 20 μl of the Dynabeads M-280 Sheep anti-Mouse IgG, and incubated for 2 h at 4°C. The beads with chromatin were washed twice with 1x RIPA (150mM) buffer, once with 1x RIPA (500mM) buffer, and once with 1x TE buffer. Sonicated chromatin equivalent to 5 x 10^4^ cells was subjected to the following DNA extraction procedures without immune-precipitation steps to obtain input DNA. After de-crosslinking, Protease K treatment, and DNA purification using Agencourt AMPure XP beads (Beckman Coulter), ChIP DNA and input DNA were eluted with 40 μl of water. Quantitative PCR was performed using 1/80 of ChIP DNA (or 1/320 of input DNA) as template DNA and the SYBR Premix Ex Taq Tli RNaseH Plus (Takara RR420A) in a 10 μl reaction volume on the 7500 Fast Real-Time PCR System (Applied Biosystems) under the following PCR cycle conditions: 95°C for 30 sec, and 40 cycles of 95°C for 5 s and 60°C for 34 s. PCR primers used are listed in S4 Table. ChIP efficiency (% input) of each ChIP DNA samples was calculated as described in the manual of LowCell# ChIP kit (Diagenode C01010070).

## Results

### Identification of a 1.8 kbp genomic segment containing a DNA methylation boundary

**Fig 1:**
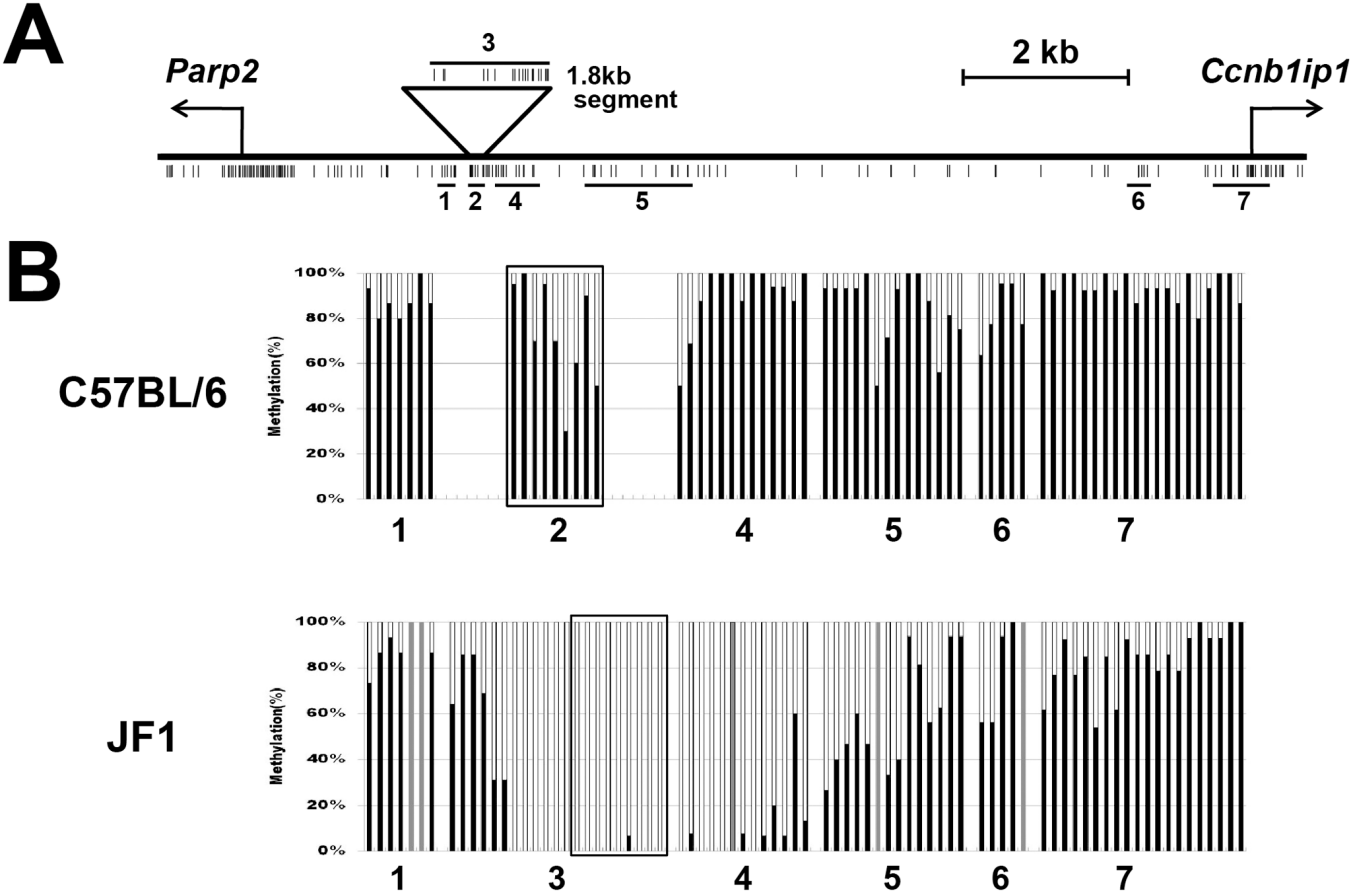
The DNA methylation boundary identified in the 1.8 kbp segment that is present in the JF1 genome but absent in the B6 genome. (A) A schematic map of the *Ccnb1ip1*-*Parp2* locus. The 1.8 kbp segment is located between the *Parp2* and *Ccnb1ip1* genes. Individual CpG sites are shown as vertical bars. Horizontal bars (numbered from 1 to 7) indicate the genomic regions whose DNA methylation levels were assessed by bisulfite sequencing. (B) DNA methylation statuses of the genomic regions #1 to #7 shown in Fig 1A in E9.5 embryos of C57BL/6 (top) and JF1 (bottom). Each black bar shows the DNA methylation level (0 to 100%) at an individual CpG site. Gray bars indicate CpG sites present in the B6 strain but absent in the JF1 strain due to DNA polymorphism. Sequence analysis revealed that the 220bp region at the right end of the 1.8kbp segment was present in the B6 genome in an inverted orientation. This 220bp interval is boxed: Region 2 in C57BL/6 and part of Region 3 in JF1.

When examining DNA methylation status of the genomic interval between *Parp2* and *Ccnb1ip1* in E9.5 embryos of C57BL/6 and JF1/Ms strains (B6 and JF1 hereafter) by bisulfite sequencing, we noticed that some regions (Regions 2, 4, 5 in Fig 1A) were differentially methylated in a strain-dependent manner (heavily methylated in B6, and less methylated or unmethylated in JF1) (Fig 1B). PCR amplification of JF1 genomic DNA using several primer pairs targeted to differentially methylated regions identified a 1.8 kbp genomic segment that was present in the JF1 strain but absent in the B6 strain (Region 3 in Fig 1A and S1 Fig). Sequence analysis revealed that this 1.8kbp segment consisted of a 1,579 bp interval deleted in the B6 genome, and the remaining 220 bp interval (boxed in Fig 1B) present in the B6 genome but in an inverted orientation. Furthermore, BLASTN analysis against the whole genome sequence (wgs) database revealed that the segment was also present in the *Ccnb1ip1* locus of the mouse inbred 129X1/SvJ strain (GenBank accession numbers: AAHY01113755.1 and AAHY01513047.1). A phylogenic analysis of 94 inbred Mouse HapMap strains including B6, JF1, and 129X1/SvJ has shown a closer distance of JF1 and 129X1/SvJ than that of JF1 and B6 (Fig 3 in [35]). Therefore, the 1.8kbp segment was estimated to have been transmitted to JF1 and 129X1/SvJ from one of the four founder strains [36] of inbred mouse strains, but to have been deleted sometime during inbreeding towards establishing the B6 strain. We subsequently determined the cytosine DNA methylation status of all CpG sites in the 1.8kb segment (Region 3 in Fig 1A) in an E9.5 embryo of the JF1 strain by bisulfite sequencing. We observed that the extent of differential methylation between B6 and JF1 is most striking in Regions 2 and 4, and the 1.8 kbp segment (Region 3) contained a distinct DNA-methylation boundary (Fig 1B).

To clarify whether these differentially methylated regions are associated with transcriptionally active and repressed chromatin states, we performed ChIP assays for H3K4me3 as an active chromatin mark and for H3K9me3 and H3K27me3 as repressed chromatin marks using chromatin prepared from E9.5 embryos of B6 and JF1 strains. We assessed the enrichment of these three histone modifications at two differentially methylated regions, Region 2 in B6 (corresponding to the boxed part in Region 3 in JF1) and Region 4 (Fig 1B), by quantitative PCR analysis, and found that these regions were not highly enriched with any of three histone modifications examined (S2 Fig). These results indicate that these differentially methylated regions between B6 and JF1 acquire high levels of DNA methylation without the involvement of H3K9me3 or H3K27me3-mediated silencing machinery in the E9.5 embryo of the B6 strain.

### Identification of an enhancer-blocking insulator element in the 1.8 kbp segment

**Fig 2:**
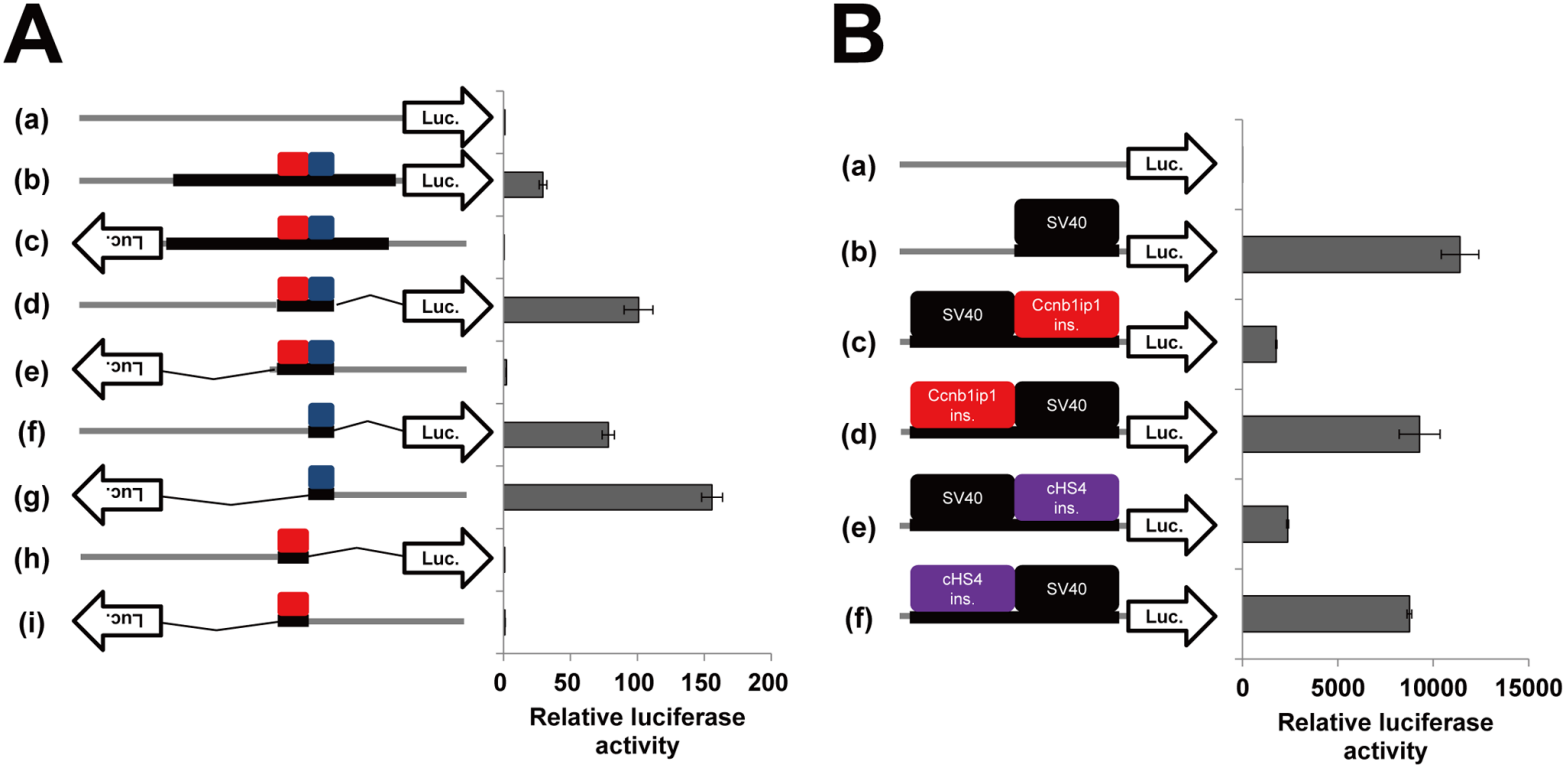
Luciferase reporter assays to evaluate the enhancer-blocking activity of the *Ccnb1ip1* insulator. (A) Identification of the *Ccnb1ip1* insulator and enhancer in the 1.8 kbp fragment. DNA fragments and their orientations cloned immediately upstream of luc2 in the pGL4.23 [luc2/minP] vector (a) were: 1.8kbp fragment in the sense orientation (b) and in the antisense orientation (c), 464-bp (nt.886 to nt.1349) fragment in the sense (d) and the antisense (e) orientations, 185-bp (nt.1128 to nt.1312) fragment in the sense (f) and the antisense (g) orientations, and 242-bp (nt.886 to nt.1127) fragment in the sense (h) and the antisense (i) orientations. Cos7 cells were transfected with these vectors and measured for the luciferase activities. Gray and black lines represent the vector backbone and cloned fragments, respectively. Red and blue boxes represent the 242 bp insulator and the 185 bp enhancer, respectively. Luciferase activities relative to that of the control vector (a) are shown (mean ± sd, n = 3). (B) Enhancer-blocking activity of the *Ccnb1ip1* insulator against the SV40 enhancer. Red, purple, and black boxes represent the *Ccnb1ip1* insulator, cHS4 insulator, and SV40 enhancer, respectively. DNA elements cloned upstream of luc2 in the pGL4.23 vector (a) were: SV40 enhancer (b), SV40 enhancer and *Ccnb1ip1* insulator in order of enhancer, insulator, and minimal promoter (c) and in order of insulator, enhancer, and minimal promoter (d), SV40 enhancer and cHS4 insulator in order of enhancer, insulator, and minimal promoter (e) and in order of insulator, enhancer, and minimal promoter (f). Luciferase activities relative to that of the control vector (a) are shown (mean ± sd, n = 3).

We hypothesized that the 1.8 kbp segment present in JF1 but absent in B6 contains an insulator element that maintains its hypomethylation status, and subjected the DNA segment to luciferase reporter assays (luc assays), in which the pGL4.23 [luc2/minP] vector carrying a minimal promoter and the luc2 gene, and Cos7 cells were used. The 1.8 kbp DNA segment enhanced luciferase expression when it was cloned immediately upstream of the minimal promoter in the vector in the sense but not in the antisense orientation (29 folds vs. 0.44 folds) (Fig 2A). To narrow down the regions responsible for the observed enhancer activity and its dependence on the cloning directions, we carried out the luc assays for various sub-regions from the 1.8 kbp segment. When a 464-bp fragment corresponding to nt.886 to nt.1349 was cloned, enhancer activity was dependent on the cloning directions (sense 100 folds vs. antisense 2.1 folds). We further divided the 464-bp fragment into a 242-bp (nt.886 to nt.1127) fragment and a 185-bp (nt.1128 to nt.1312) fragment. The 242-bp fragment had no enhancer activities, but the 185-bp fragment showed enhancer activities regardless of its cloning directions. Similar results were also obtained in the luc assays using NIH3T3 and HeLa cells (S3 Fig). These results suggest that the 185-bp region contains an enhancer element, whose activity is bidirectional (in the absence of the adjacent insulator), and that the 242-bp region contains an insulator element, which limits the functional direction of the adjacent enhancer element.

In E9.5 embryos, *Parp2* expression levels were similar between B6 and JF1 strains; however, *Ccnb1ip1* expression level in JF1 was apparently higher (47.0-folds) than that in B6 (S4 Fig). Such expression patterns agree with the putative functions of the 242-bp and 185-bp regions revealed by the luc assays: the putative 185-bp enhancer may be contributing to a much higher expression level of *Ccnb1ip1* in JF1 compared to B6, and if so, the putative 242-bp insulator may be preventing the action of the enhancer to the *Parp2* promoter in JF1. We therefore designated these putative *cis*-regulatory elements as the *Ccnb1ip1* enhancer and the *Ccnb1ip1* insulator, respectively.

We subsequently investigated whether the *Ccnb1ip1* insulator shows an enhancer-blocking activity against an exogenous enhancer, the SV40 enhancer. The SV40 enhancer and the *Ccnb1ip1* insulator were cloned in tandem upstream of the minimal promoter in the luciferase vector, and subjected to luc assays using Cos7 cells. Consistent with its position-dependent effect against the *Ccnb1ip1* enhancer (Fig 2A), the *Ccnb1ip1* insulator showed a remarkable enhancer-blocking activity, when it was cloned between the SV40 enhancer and the minimal promoter (Fig 2B). We used the 250-bp core sequence of the cHS4 insulator, known to be one of the most effective insulators, as a control DNA fragment for enhancer-blocking activity. The enhancer-blocking activity of the *Ccnb1ip1* insulator against the SV40 enhancer was slightly higher than that of the cHS4 insulator (85% reduction vs 79% reduction). Taken together, the 242-bp region within the 1.8 kbp genomic segment, designated as the *Ccnb1ip1* insulator, exhibited enhancer blocking activities against the *Ccnb1ip1* enhancer as well as the SV40 enhancer.

### Barrier activity of the *Ccnb1ip1* insulator

**Fig 3:**
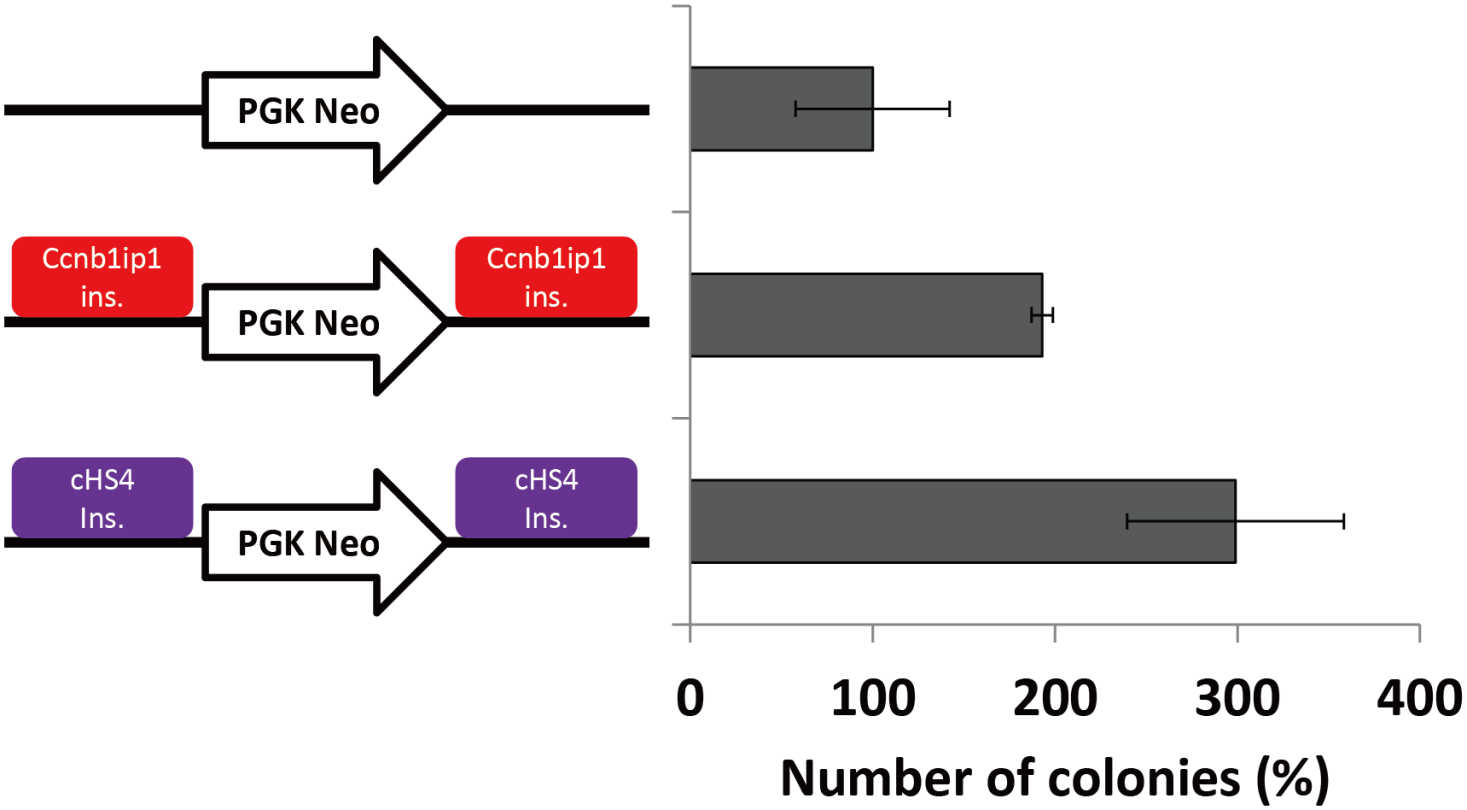
Colony formation assays to evaluate the barrier activity of the *Ccnb1ip1* insulator. The *Ccnb1ip1* insulator or the cHS4 insulator was cloned at both sides of the neomycin-resistant gene (PGK-neo). Red and purple boxes represent the *Ccnb1ip1* insulator, and cHS4 insulator, respectively. Cos7 cells were transfected with these vectors in triplicates. The average numbers of the colonies formed without G418 selection are 86, 72, and 82, for PGK-neo alone, PGK-neo with *Ccnb1ip1* insulators, and PGK-neo with cHS4 insulators, respectively. The average numbers of the colonies formed with G418 selection are 9, 14, and 24, for PGK-neo alone, PGK-neo with *Ccnb1ip1* insulators, and PGK-neo with cHS4 insulators, respectively. Percentage numbers of the G418-resistant colonies relative to that for PGK-neo alone are shown for PGK-neo with *Ccnb1ip1* insulators and PGK-neo with cHS4 insulators (mean ± sd, n = 3).

It has been reported that the cHS4 insulator has a barrier activity that protects a gene from silencing by DNA methylation [10,12,25,27,29]. To evaluate whether the *Ccnb1ip1* insulator also shows barrier activity, we subjected it, as well as the 250-bp core sequence of the cHS4 insulator, to colony-formation assays. The *Ccnb1ip1* insulator or the cHS4 insulator was cloned at both sides of the neomycin-resistant gene (PGK-neo), and each of the vector constructs was transfected into Cos7 cells in triplicates. At 12h after transfection, 4,000 cells each were subjected to colony formation in a 100mm dish with or without G418 selection. After 13 days, the numbers of colonies were counted. Compared to the PGK-neo alone, the presence of the *Ccnb1ip1* insulators and the cHS4 insulators at both sides of the PGK-neo cassette increased the numbers of the G418-resistant colonies roughly twice and three times, respectively (Fig 3). These results demonstrate that, although it was weaker than that of the cHS4 insulator, the *Ccnb1ip1* insulator exhibited a barrier activity in colony formation assays.

### Evolutionary conservation of the *Ccnb1ip1* gene structure and of the enhancer blocking activity of the *Ccnb1ip1* insulator among mammalian species

**Fig 4:**
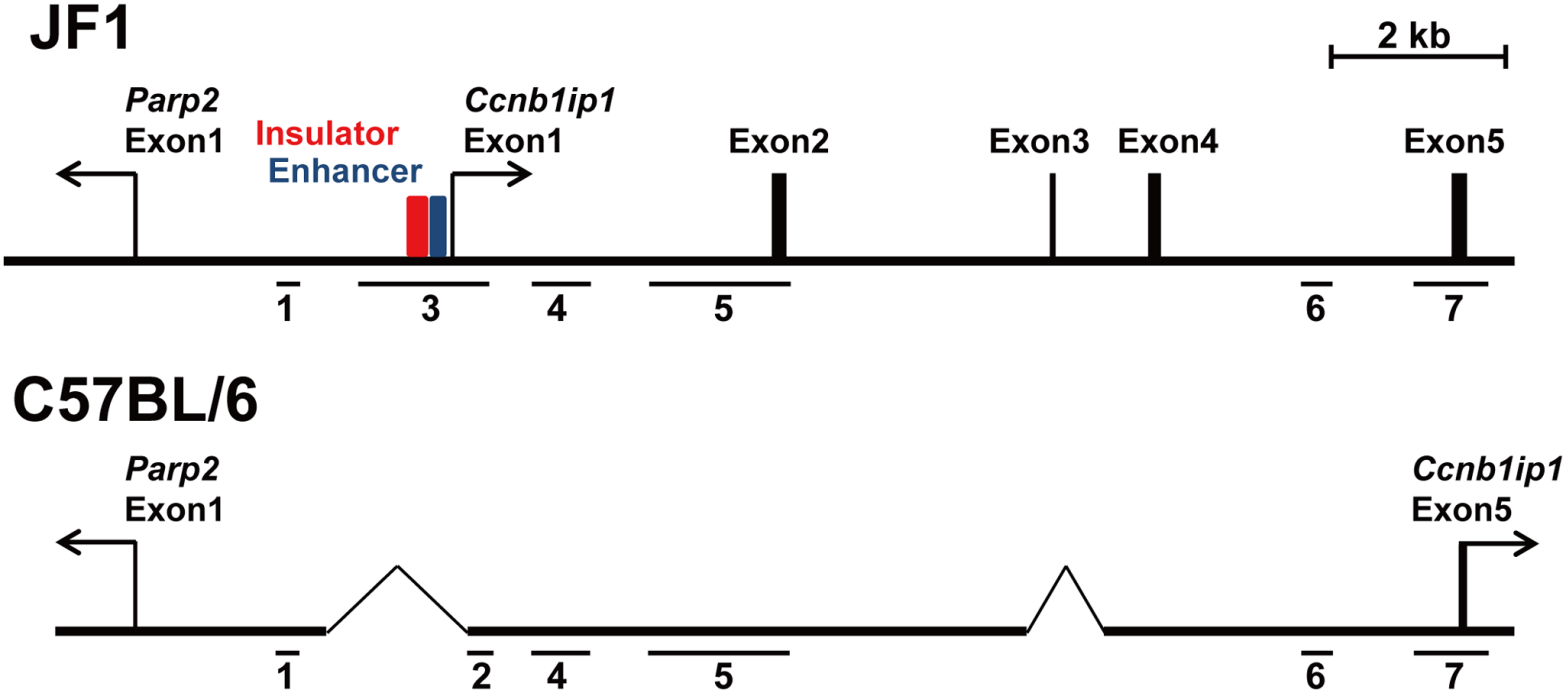
Exon-intron structure of the *Ccnb1ip1* gene in the JF1 and C57BL/6 genomes. RT-PCR products from exon 1 to exon 5 were detected in an E9.5 embryo of JF1. Exon 1 of *Ccnb1ip1* was located in the vicinity of the *Ccnb1ip1* insulator (red box) and enhancer (blue box) in the JF1 genome. The sequences corresponding to exon 2 and exon 4 of the *Ccnb1ip1* gene were found to be present, but those corresponding to exon 1 and exon 3 to be absent (deleted) in the B6 genome. The horizon bars (1 to 7) indicate genomic regions for which DNA methylation statuses were assessed (Fig 1).

We examined whether the mouse *Ccnb1ip1* insulator is evolutionarily conserved among other species. Whereas the mouse reference (B6) genome lacks the *Ccnb1ip1* insulator sequence, we found a genomic region highly similar to the 242-bp insulator sequence at the human *CCNB1IP1* locus. Further extensive sequence homology searches using BLAST revealed that the *Ccnb1ip1* insulator is conserved in a wide variety of mammalian species including humans, chimps, orangutans, rhesus macaques, marmosets, cats, horses, rabbits, and rats (69%, 70%, 71%, 68%, 68%, 70%, 67%, 68%, and 84% identical, respectively) (S5 Fig). In the human and rat genomes, the transcriptional start site of the *CCNB1IP1* gene was found to be located within 300 bp from the region highly similar to the mouse insulator sequence (S6 Fig). These results suggested that the genomic organization observed in the human and rat *CCNB1IP1* locus is also conserved in the JF1 and 129X1/SvJ mouse strains. In fact, pairwise alignment of the human *CCNB1IP1* mRNA sequence (GenBank accession number NM_021178) with mouse genomic sequences detected a region similar to human exon 1 in the 1.8 kbp sequence (S1 Fig) present in the JF1 stain and regions similar to human exons 2–4 in the 129X1/SvJ genomic sequences AAHY01113755.1 and AAHY01513047.1. We confirmed the existence of these putative exons in the JF1 strain by performing RT-PCR, whose forward and reverse primers were targeted to the exon 1/2 junction and exon 5, respectively. Direct sequencing of the RT-PCR products and sequence alignment analysis using BioEdit (http://www.mbio.ncsu.edu/bioedit/bioedit.html) revealed that the sequences of exons 1, 2, 3 and 4 (Fig 4) are highly conserved among humans, rats, and mice (S7 Fig) The RT-PCR products were detected in E9.5 embryo of the JF1 strain but not in that of the B6 strain (data not shown). These results demonstrate that the mouse *Ccnb1ip1* insulator and enhancer are located immediately upstream of the transcriptional start site of the *Ccnb1ip1*gene in the JF1 strain, and that these *cis*-elements as well as the exon 1 of the *Ccnb1ip1* gene were deleted in the B6 strain. In E9.5 embryos of the B6 strain, *Ccnb1ip1* expression was detectable when RT-PCR primers (S4 Table) targeted to the second last and the last (the 3’ UTR) exons of the gene were used (S4 Fig), indicating the existence of an alternative promoter other than the deleted exon 1 in the B6 strain.

**Fig 5:**
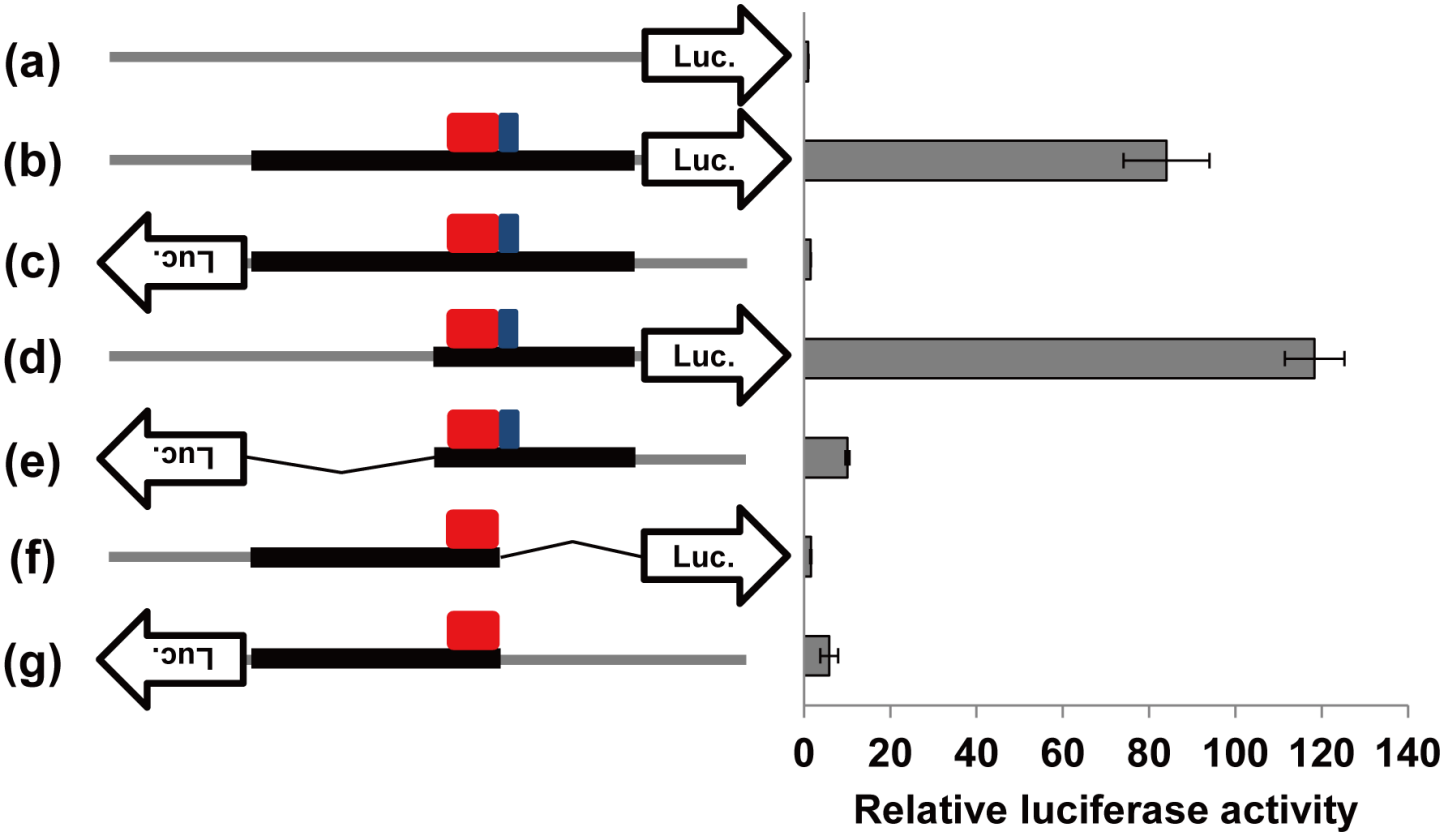
Luciferase reporter assays to evaluate of the enhancer-blocking activity of the human putative *CCNB1IP1* insulator. The 1,878 bp fragment that contains the human sequences homologous to the mouse *Ccnb1ip1* insulator and enhancer was cloned in the pGL4.23 [luc2/minP] vector (a) in the sense (b) and the antisense (c) orientations. Two partial fragments, a 975 bp fragment containing both putative insulator and enhancer, and a 1,207 bp fragment containing only the putative insulator, were also cloned in the sense (d, f) and the antisense (e, g) orientations. Cos7 cells were transfected with these vectors and measured for the luciferase activities. Gray and black lines represent the vector backbone and cloned fragments, respectively. Red and blue boxes represent the putative *CCNB1IP1* insulator (260 bp) and enhancer (66bp), respectively. Luciferase activities relative to that of the control vector (a) are shown (mean ± sd, n = 3).

To investigate whether the human genomic segment positionally orthologous to the mouse *Ccnb1ip1* insulator exhibits insulator and enhancer activities, the corresponding 1,878 bp DNA fragment was subjected to luc assays (Fig 5). By the alignment of human and mouse genomic DNA sequences, a 260 bp and a 66 bp intervals were identified as putative insulator (S4 Fig) and enhancer (data not shown) sequences, respectively, in the 1,878 bp human sequence. The human putative enhancer region was shorter because it lacked the CT-rich repeat region present in the mouse *Ccnb1ip1* enhancer region (S1 Fig). When the fragment was cloned in the sense orientation in the pGL4.23 [luc2/minP] vector, luciferase expression was enhanced (84.0-folds). On the other hand, such an enhancer activity was not detected when the sequence was cloned in the antisense orientation. When a 975 bp fragment (nt.1 to nt.975 in the 1,878 bp sequence) containing putative *CCNB1IP1* insulator and enhancer was cloned in the vector, its enhancer activity was dependent on the cloning direction (118.4-folds in the sense and 10.1-folds in the antisense orientation). On the other hand, when a 1,207 bp (nt.672 to nt.1878) fragment containing only putative *CCNB1IP1* insulator but not the putative enhancer was subjected to the luc assays, the fragment did not affect the luciferase expression level much (1.6-folds in the sense and 5.9-folds in the antisense orientation). These results indicate that the human 1,878 bp genomic segment contains a region functionally orthologous to the mouse *Ccnb1ip1* insulator/enhancer region. The observed evolutionary conservation of the *Ccnb1ip1* insulator strongly suggests its endogenous role in the transcriptional regulation of the *Ccnb1ip1* gene.

## Discussion

In this study, we identified putative insulator and enhancer elements located at the upstream region of the mouse *Ccnb1ip1* gene, and revealed that these elements were deleted in the C57BL/6 strain but present in the JF1 strain. The putative *Ccnb1ip1* insulator (242-bp in size) exhibited enhancer blocking activities against the putative *Ccnb1ip1* enhancer (185-bp in size), which is located adjacently to the insulator in the JF1 genome, and the SV40 enhancer in luciferase reporter assays using Cos7 cells. It should be also noted that the enhancer blocking activity of the putative *Ccnb1ip1* insulator against the SV40 enhancer was comparable to that of the cHS4 insulator, which is located in the chicken β-globin locus and known to be one of the most effective insulators. Sequence analysis of the *Ccnb1ip1* locus in a wide variety of mammalian species revealed that the *Ccnb1ip1* insulator sequence is highly conserved among them (S5 Fig). The putative human *CCNB1IP1* insulator was confirmed to show an enhancer blocking activity against its adjacent enhancer in reporter assays (Fig 5). While experimental evidence has been limited to artificial reporter assays, such sequence and functional conservation of the insulator element among mammalian species suggests its endogenous role in gene regulation. Furthermore, the expression patterns of *Parp2* and *Ccnb1ip1* in E9.5 embryos of B6 and JF1 strains (S4 Fig) fit with the postulation that the putative insulator and enhancer are involved in the transcriptional control at the *Parp2*-*Ccnb1ip1* locus *in viv*o: the enhancer acts only on the *Ccnb1ip1* promoter, but not the *Parp2* promoter due to the intervention of the insulator between them. Based on these lines of direct and indirect evidence, we designated the identified putative *cis*-elements as the *Ccnb1ip1* insulator and the *Ccnb1ip1* enhancer.

We also demonstrated that the *Ccnb1ip1* insulator exhibited a barrier activity in the colony formation assays using the PGK-neo cassette as a reporter in Cos7 cells. The *Ccnb1ip1* insulator may help develop vectors for stable expression of transgenes. However, the barrier activity of the *Ccnb1ip1* insulator was weaker than that of the cHS4 insulator. It has been reported that the cHS4 insulator can be separated into both enhancer-blocking and barrier insulators [8]. Further characterization of the *Ccnb1ip1* insulator to pinpoint enhancer-blocking and barrier elements within it may help identify a DNA fragment possessing a stronger barrier activity.

CTCF and cohesin are known as insulator-binding proteins [37,38]. Genome-side distribution of these proteins has been identified by chromatin immunoprecipitation-sequencing (ChIP-seq) studies [39–41]. However, CTCF and cohesin were found to be unbound to the human putative *CCNB1IP1* insulator region in these ChIP-seq studies (S8 Fig). On the other hand, a paired-end tag sequencing (ChIA-PET) dataset for long-range chromatin interactions associated with RNA polymerase II in human cells [42] demonstrated that the region containing putative *CCNB1IP1* insulator interacts with the region containing the transcription start site (TSS) of *PARP2*, which is 10kb away from the *CCNB1IP1* insulator intra-chromosomally, and the region containing the TSSs of *OSGEP* (encoding O-sialoglycoprotein endopeptidase) and *APEX1* (encoding apurinic-apyrimidinic endonuclease 1) genes, which is 121 kb away intra-chromosomally. These results suggest the possibility that the *CCNB1IP1* insulator functions in a CTCF- and cohesin-independent manner, and is involved in the formation of long-range chromatin loops associated with RNA polymerase II. An insulator identified at immediate upstream of the *ANK1* promoter [18] is another example of CTCF-independent insulators (S8 Fig). Further characterization of these promoter-associated and CTCF-unbound insulators may facilitate better understanding of the roles of insulators in higher-order chromatin organization and transcription regulation.

We examined the chromatin states of the human *CCNB1IP1* promoter region in seven cell types using the ENCODE data [40] (S9 Fig). The putative insulator and enhancer regions were generally classified as part of active promoter/enhancer regions by ChromHMM, a computational tool for chromatin state segmentation. The putative enhancer (66 bp) and exon 1 of *CCNB1IP1* but not the putative insulator tended to be overlapped with ChIP-seq peaks for RNA polymerase II. The peak heights of transcriptionally active chromatin marks, H3K4me3 and H3K27ac, varied among seven cell types, but tended to be lower or absent in the insulator/enhancer regions, abruptly become higher at immediate downstream of the enhancer region, and last for nearly 1kb, demonstrating distinct chromatin states between the putative insulator/enhancer elements and the promoter at the human *CCNB1IP1* locus (S9 Fig). Transcriptionally repressed chromatin marks, H3K9me3 and H3K27me3, were absent in all of the insulator, enhancer, and promoter regions. These histone modification patterns observed in human cells are mostly consistent with those in the mouse E9.5 embryos (S2 Fig). Because the first exon of the *Ccnb1ip1* gene in the mouse JF1 strain was identified immediately downstream of the insulator and enhancer elements (Fig 4, S7 Fig) Although the 101 bp interval in Region 2 (the right edge of Region 3 in JF1) and/or the 118 bp interval in Region 4 subjected to ChIP-qPCR (S2 Fig) were expected to be enriched with H3K4me3 because of their proximity to a putative transcriptional start site of *Ccnb1ip1* (Fig 4), the enrichment levels of H3K4me3 at these intervals were apparently lower than that of the *Gapdh* promoter region. The low level of *Ccnb1ip1* expression (relative to *Gapdh* expression) in E9.5 embryos in JF1 may account for the observed levels of H3K4me3 enrichment. Despite the hypermethylation at CpG sites within Region 2 and Region 4 in the E9.5 embryo of the B6 strain (Fig 1B), the regions were not enriched with repressed chromatin marks (S2 Fig). While concomitance of such DNA methylation and histone modification patterns is seemingly discrepant, a recent large scale study for epigenome profiles [43] has demonstrated that 67.8% of the human genome possesses such an epigenetic state.

The *Ccnb1ip1* gene encodes an ubiquitin E3 ligase, which functions in the progression of the cell cycle through G2/M [21] and is considered to be involved in tumor development. Loss-of-function mutation in the mouse *Ccnb1ip1* gene is also shown to disrupt meiotic crossing-over [20]. The human CCNB1IP1 protein has been reported to interact with merlin, a tumor suppressor protein [22], and the expression levels of the *CCNB1IP1* gene have been found to be altered in cancers such as uterine leiomyoma [44], melanoma [45], and breast cancer [24]. Therefore, further characterization of the insulator and enhancer in the *Ccnb1ip1* locus may help understand the roles of CCNB1IP1 in cell cycle regulation and tumor development.

## Supporting Information

S1 Fig The DNA sequence of the 1,799bp fragment located in between *Parp2* and *Ccnb1ip1* genes identified in the JF1 genome. The 242 bp *Ccnb1ip1* insulator and the 185 bp *Ccnb1ip1* enhancer are highlighted in red and blue, respectively. Sequence analysis revealed that the 1,579 bp interval (from nt.1 to nt.1,579) was deleted in the B6 genome, and that the remaining 220 bp interval (from nt.1,580 to nt.1,799, boxed) was present in the B6 genome but in an inverted orientation.
(DOCX)

S2 Fig ChIP-qPCR analysis of the Ccnb1ip1 insulator, Region 2, and Region 4 for histone modifications (H3K4me3, H3K9me3, H3K27me3) in the E9.5 embryos of B6 and JF1 strains. The positions of the PCR amplicons are shown (left). ChIP efficiencies (% input) of six genomic regions assessed are shown (right). *Gapdh* promoter, *Snord* cluster, and *Uncx* regions were analyzed as positive regions for H3K4me3, H3K9me3, H3K27me3, respectively. The sequences of the PCR primers used are shown in S4 Table.
(DOCX)

S3 Fig Luciferase reporter assays to evaluate the enhancer-blocking activity of the *Ccnb1ip1* insulator in NIH3T3 (A) and HeLa (B) cells. Gray and black lines represent the vector backbone and cloned fragments, respectively. Red and blue boxes represent the 242 bp *Ccnb1ip1* insulator and the 185 bp enhancer, respectively. Luciferase activities relative to that of control vector are shown (mean ± sd, n = 3).
(DOCX)

S4 Fig Expression levels of *Ccnb1ip1* (A) and *Parp2* (B) in E9.5 embryos of B6 and JF1 strains determined by quantitative RT-PCR. *Ccnb1ip1* and *Parp2* expression levels normalized by the *Gapdh* expression level are shown (mean ± sd, n = 3). Vertical axis represents the expression level relative to that of *Gapdh*.
(DOCX)

S5 Fig Homology of the mouse *Ccnb1ip1* insulator DNA sequence with orthologous sequences in humans, chimps, orangutans, rhesus macaques, marmosets, cats, horses, rabbits, and rats. Conserved sequences are highlighted.
(DOCX)

S6 Fig Location of the genomic region homologous to the mouse *Ccnb1ip1* insulator sequence at the *PARP2-CCNB1IP1* locus in the rat (upper) and human (lower) genomes. The position of the homologous sequence is indicated by a vertical bar.
(DOCX)

S7 Fig Alignments of human, mouse (JF1), and rat sequences of exons 1 to 4 of the *Ccnb1ip1* gene. Sequences for each exon were aligned using BioEdit (http://www.mbio.ncsu.edu/bioedit/bioedit.html). Conserved sequences are highlighted.
(DOCX)

S8 Fig Occupancy patterns of CTCF at *HBB* (A), *CCNB1IP1-PARP2* (B), and *ANK1*(C) loci in various cell lines. CTCF ChIP-seq data from the ENCODE project (https://genome.ucsc.edu/ENCODE/) are shown. Red rectangles indicate the location of an insulator for each locus: 5’HS4 insulator at *HBB* locus, the putative *CCNB1IP1*-insulator, and the *ANK1* insulator [18]. Whereas CTCF peaks were observed in the majority of the cell lines at the 5’HS4 insulator, no peaks were observed at *CCNB1IP1* and *ANK1* insulators.
(PPTX)

S9 Fig Chromatin states and histone modification patterns at the human *CCNB1IP1* promoter region in seven cell types. “Histone Modifications by ChIP-seq” and “Chromatin State Segmentation by HMM” tracks of ENCODE/Broad Institute data (http://genome.ucsc.edu/ENCODE/) are shown for GM12878, H1-hESC, K562, A549, HeLa-S3, HepG2, and HUVEC cells.
(PPTX)

S1 Table Primers for bisulfite sequencing analysis. (XLSX)

S2 Table Primers for vector construction for reporter assays. (XLSX)

S3 Table Primers for vector construction for colony assay. (XLSX)

S4 Table Primers for quantitative RT-PCR and ChIP-PCR. (XLSX)
