# Supplementary material for: An Insulator Element Located at the Cyclin B1 Interacting Protein 1 Gene Locus Is Highly Conserved among Mammalian Species
Source: PLoS One. 2015 Jun 25;10(6):e0131204. doi: 10.1371/journal.pone.0131204 (PMC4481373; doi:10.1371/journal.pone.0131204)
Supplement: S1 Fig — The 242 bp Ccnb1ip1 insulator and the 185 bp Ccnb1ip1 enhancer are highlighted in red and blue, respectively. Sequence analysis revealed that the 1,579 bp interval (from nt.1 to nt.1,579) was deleted in the B6 genome, and that the remaining 220 bp interval (from nt.1,580 to nt.1,799, boxed) was present in the B6 genome but in an inverted orientation. (DOCX) [file pone.0131204.s001.docx]

GGATCAATCCACCTCCCTTTTCCTCCCCAGTGCCAGGATTAAAAGTGTGTGCCACCTTTCCTGGTTCATAATGCCTTTTCATTTATTTATTTTTAATAGATGAGTGATATTCCATTGTATAGTACCACATTTTTTTTCCTGATCCATTTATCTCTTGATGGATGTCTAGGCTATTTCTAATTCCTGGCTATTATGTACAGCAACAGTGACCATGGATGAACAAGTGCCTTTGTAGCAGACAGACACATCCTTTGGGTGTATGCCTAAGAGTGGTATACAGCTGGAATCTGAGGAGACTCTGTTCCCAGCTTCCTGAGGAATGCACAGTGATTTCCACAGTGGCTGTGCAAGCTTTCACTTCCCCAGTAATAGACGCATGTTTCCCTTTCCCTACAGCTTTGCCAGCATTGTTCTATTTGTTCTATTGATCTTGGTCCTCTAATGTGGGTAAGGTGGAGTAGTTTGGATTGGCATTTCCCTGATAACTACGGATGCTGACCATTGCTTCGGTGATCCTGAATCATTTGCCTTTCATCCTTTGAGATTTCTTTGTTTAGATATGGGATATTCATCTTCTTGATGTGCAGTTTTTAAAGCTTTGAATGTATTTTAGATATTAATCCTCTATTGGAATGTACTTGGTAAAGATCTTTTCCCATTCTATAAAGTGCTTTTTTTTTTTTTGTCTGAATGATGGTGTTTGCTATACAGATGCCACATAAAGTTTATATCAAGGGCCAACCTGTTAACCCAACCCTTGTTGTACTACAATATTTTAAGTTGGGGGAATGAAGCCTTAGATGTGTACAAGTATTCAAATTTTATCTTGAAGGATAAGCATTAAACCACCCATTAATAATTGTATTGTCTGTTAGCTTGAAGGTTGCCCTGAAGGCAATTTCTAAGTTGGATAGTTACAGACTGTTTATAATTGGACCTATTATTACCTTGCATAAGTTAGCTATGTTGAGGTTGCAAAAGGACGGGAGGTTACTCAGGATGGAAACTGTTTGGAATAGAACAAGGACATGCCCAAACCGTTTCAATTATCTGTGTTTGTTTGTTTTGTGTCATGATCCTGAAAGAAATTTTGTGTAGGAGTTCAATCTTAAAATGAGCGTTGAGTTGCAGTCTGGCAGCACATTTTAAAATTAAGAGGGGAACAGGGAGGAGGGGGAGAAGAAGGAGGAAGAAAAAGAAGAAGAGGAGGAGGAAGAGGAAGAAGAGGAGGAGGAAGAGGAAGAAGAGGAGGAGGAGGAGGAGGAGGAGGAGGAGGAGGAGGAGGAGGAGATCTTGAACTCAGCCAATCAGGTTCAGAGGCCCATTACTTAGGTAGCTTCCCGTCCTTGAACCTTCTCTATCTCTCGCTTCTGATGGTCTGGTAAGTGCACAGTTAATTTGGATCTCTGGTTTTTAACTCGGATCACTGGTTTTTAATTTCTCATTTTCTTGAGGGGTCTAACCGAGGGAAAGACTTTTTTTTTTTCATGTCAGCGCTGGAGTTTGTGAGTTGAGAAGAGATACGTTTCTGCTTTGGGAGGTTAGGGGAGGGCAGGCAGGTGGAGTACCTGGTTTGGTCCACGTGGGCCTCGGCAGTCCTTGTTTGCCAGCTTTCCCCAGACCAGGTGACCCAGAAACAATACATTCTGCCTCGGGCACAGTGTTCCTGAATCTGGGGAAATCCGTCTGTATTCTTTTTTTTTTTTTTTTTTTTTTTTTTCTAATTGGGAAAGGCACCGTTACTACCCCGGCGCTAACCTGGGCGGTCTCGGAGGGTGAGTGCAGCCTCTGTCTTTGGT

**S1 Fig. The DNA sequence of the 1,799bp fragment located in between *Parp2* and *Ccnb1ip1* genes identified in the JF1 genome.** The 242 bp *Ccnb1ip1* insulator and the 185 bp *Ccnb1ip1* enhancer are highlighted in red and blue, respectively. Sequence analysis revealed that the 1,579 bp interval (from nt.1 to nt.1,579) was deleted in the B6 genome, and that the remaining 220 bp interval (from nt.1,580 to nt.1,799, boxed) was present in the B6 genome but in an inverted orientation.
