# Supplementary material for: An Insulator Element Located at the Cyclin B1 Interacting Protein 1 Gene Locus Is Highly Conserved among Mammalian Species
Source: PLoS One. 2015 Jun 25;10(6):e0131204. doi: 10.1371/journal.pone.0131204 (PMC4481373; doi:10.1371/journal.pone.0131204)
Supplement: S2 Fig — The positions of the PCR amplicons are shown (left). ChIP efficiencies (% input) of six genomic regions assessed are shown (right). Gapdh promoter, Snord cluster, and Uncx regions were analyzed as positive regions for H3K4me3, H3K9me3, H3K27me3, respectively. The sequences of the PCR primers used are shown in S4 Table. (DOCX) [file pone.0131204.s002.docx]

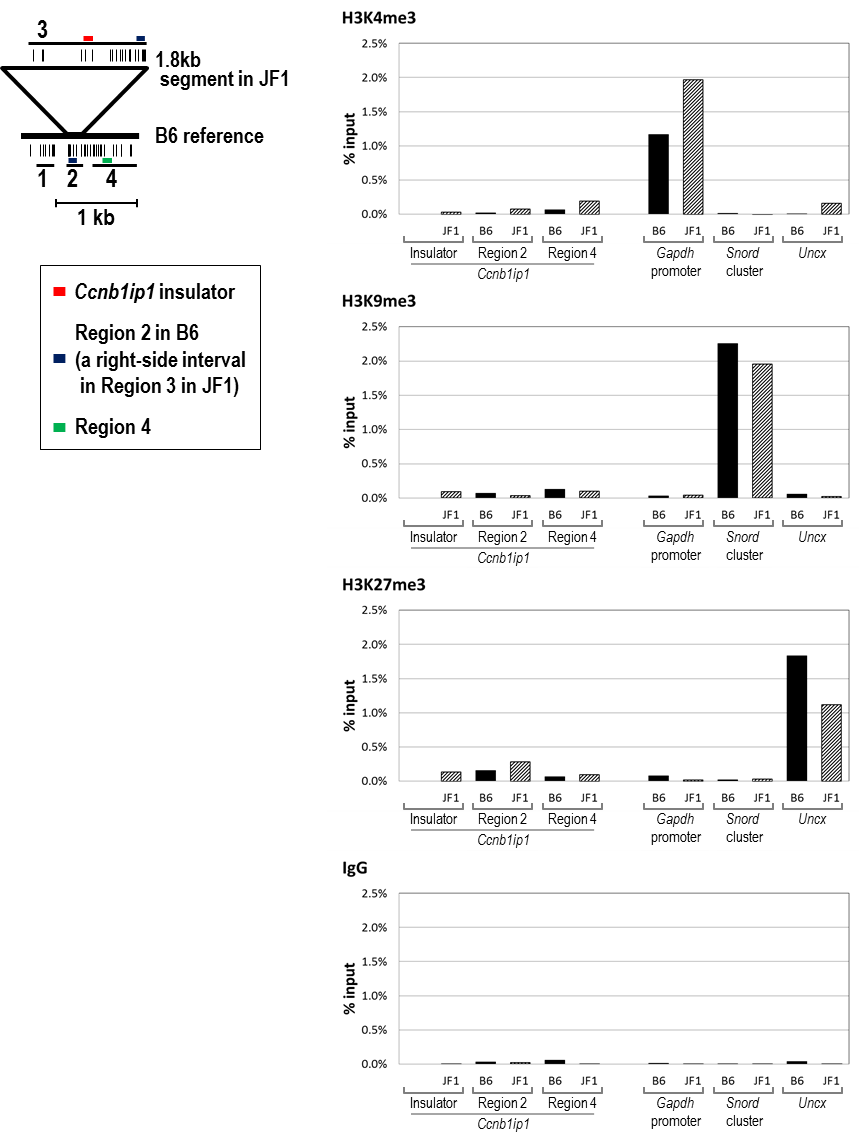


**S2 Fig. ChIP-qPCR analysis of the *Ccnb1ip1* insulator, Region 2, and Region 4 for histone modifications (H3K4me3, H3K9me3, H3K27me3) in the E9.5 embryos of B6 and JF1 strains.** The positions of the PCR amplicons are shown (left). ChIP efficiencies (% input) of six genomic regions assessed are shown (right). *Gapdh* promoter, *Snord* cluster, and *Uncx* regions were analyzed as regions positive for H3K4me3, H3K9me3, H3K27me3, respectively. The sequences of the PCR primers used are shown in S4 Table.
