# Supplementary material for: An Insulator Element Located at the Cyclin B1 Interacting Protein 1 Gene Locus Is Highly Conserved among Mammalian Species
Source: PLoS One. 2015 Jun 25;10(6):e0131204. doi: 10.1371/journal.pone.0131204 (PMC4481373; doi:10.1371/journal.pone.0131204)
Supplement: S3 Fig — Gray and black lines represent the vector backbone and cloned fragments, respectively. Red and blue boxes represent the 242 bp Ccnb1ip1 insulator and the 185 bp enhancer, respectively. Luciferase activities relative to that of control vector are shown (mean ± sd, n = 3). (DOCX) [file pone.0131204.s003.docx]

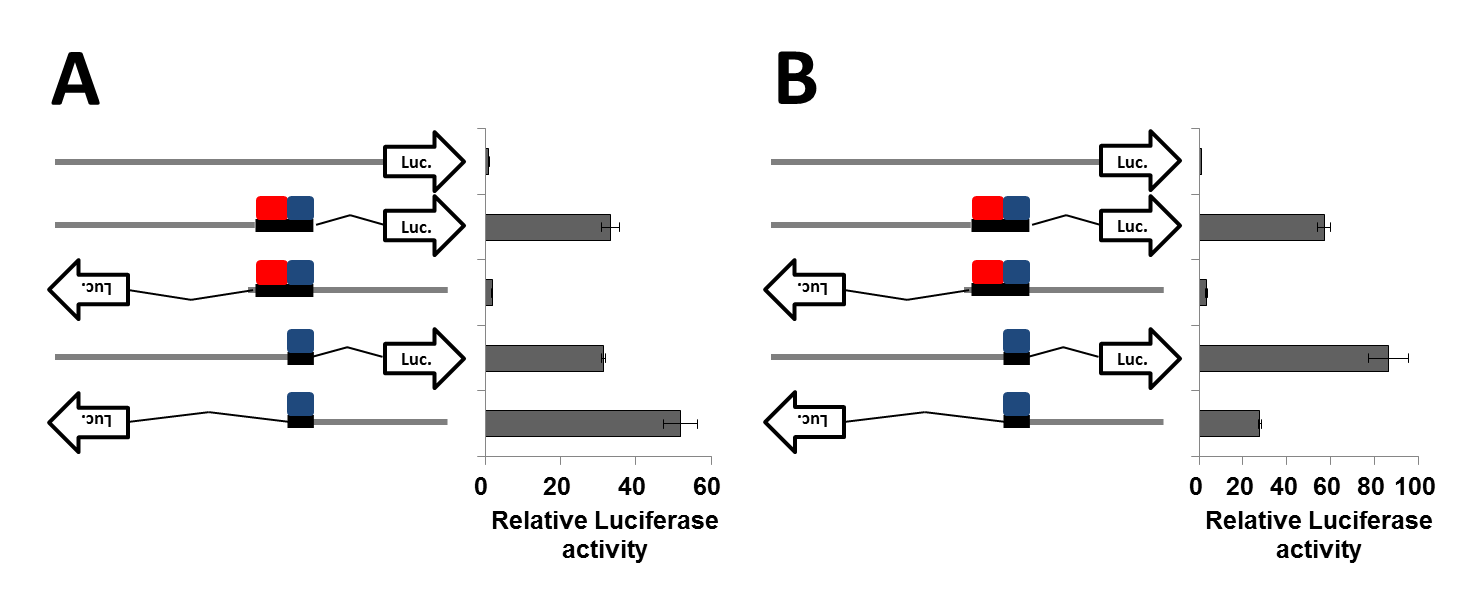


**S3 Fig. Luciferase reporter assays to evaluate the enhancer-blocking activity of the *Ccnb1ip1* insulator in NIH3T3 (A) and HeLa (B) cells.** Gray and black lines represent the vector backbone and cloned fragments, respectively. Red and blue boxes represent the 242 bp *Ccnb1ip1* insulator and the 185 bp enhancer, respectively. Luciferase activities relative to that of control vector are shown (mean ± sd, n = 3).
