# Supplementary material for: An Insulator Element Located at the Cyclin B1 Interacting Protein 1 Gene Locus Is Highly Conserved among Mammalian Species
Source: PLoS One. 2015 Jun 25;10(6):e0131204. doi: 10.1371/journal.pone.0131204 (PMC4481373; doi:10.1371/journal.pone.0131204)
Supplement: S4 Fig — Ccnb1ip1 and Parp2 expression levels normalized by the Gapdh expression level are shown (mean ± sd, n = 3). Vertical axis represents the expression level relative to that of Gapdh. (DOCX) [file pone.0131204.s004.docx]

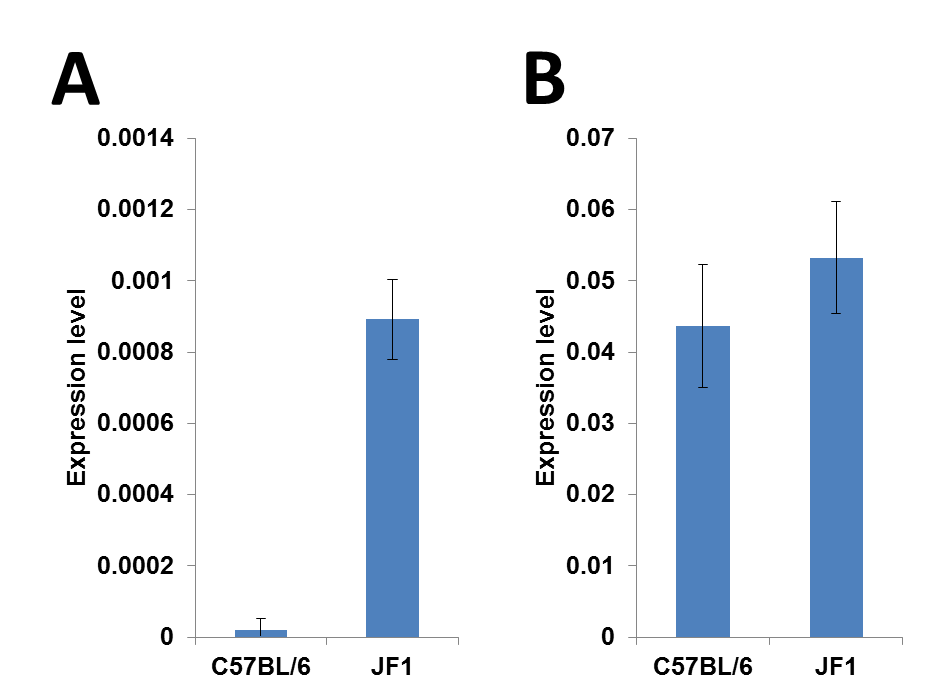


**S4 Fig. Expression levels of *Ccnb1ip1* (A) and *Parp2* (B) in E9.5 embryos of B6 and JF1 strains determined by quantitative RT-PCR.** *Ccnb1ip1* and *Parp2* expression levels normalized by the *Gapdh* expression level are shown (mean ± sd, n = 3). Vertical axis represents the expression level relative to that of *Gapdh.*
