# Supplementary material for: An Insulator Element Located at the Cyclin B1 Interacting Protein 1 Gene Locus Is Highly Conserved among Mammalian Species
Source: PLoS One. 2015 Jun 25;10(6):e0131204. doi: 10.1371/journal.pone.0131204 (PMC4481373; doi:10.1371/journal.pone.0131204)
Supplement: S5 Fig — Conserved sequences are highlighted. (DOCX) [file pone.0131204.s005.docx]

10 20 30 40 50 60 70 80 90 100

....|....|....|....|....|....|....|....|....|....|....|....|....|....|....|....|....|....|....|....|

Human AAAATGAAGGTAAATTTTAACTTGAATCGATCTAGACTGTTTATAATGGAATCTGTTG---CCTAGCATAAGTTA--AGCATGTTGAGGTCACAAA-GGA

Chimp AAAATGAAGGTAAATTTTAACTTGAATCGATCTAGACCGTTTATAATGGAATCTGTTG---CCTAGCATAAGTTA--AGCATGTTGAGGTCACAAA-GGA

Orangutan AAAATGAAGGTAAATTTTAACTTGAATCGATCTAGACTGTTTATAATGGAATCTGTTG---CCTAGCATAAGTTA--ACCATGTTGAGGTCACAAA-GGA

Rhesus AAAATGAAGGTAAATTTTAACTTGAATC-ATCTAGACTGTTTATAATGGAATCTGTTG---CCTAGCATAAATTA--ACCATGTTGAGGTCACAAA-GGA

Marmoset AAAATGAAGGTAAATTTTAACTTGAATAGATCTAGACTGTTTATAATGGAATTTGTTG---CCTAGCATCAATTGTGACCATACTGAGGTCACAAA-GGA

Cat ACCCTGAAGGTAAGTTCTAATTTGAATGGATCTAGACTGTTTATAATGAAACCTGTTA---CTTTGCATAAATTA--ACCCTATTGAAGTC-CAAA-GGT

Horse AGCCTGAAGGTAGGTTTTAACTTGAATAGACCTAGTCTGTT-ATAATGAAACCTGTTA---CCTTGCATAAATTA--ACCATATGGAGGTCATAAA-GGA

Rabbit -CTCTGAAGCCTAGTTCTAACTTGAATGGGTCTAGAATCTTTATAATGGAAGCCGTTA---TCTTACATAACTCA---CCATGTTCAGATTACAAA-ACA

Rattus GCCCTGGATACGATTTCTGTCTTGGATTCTTATGGACTGTTTATAATTGGCCCTATTA---CCTTGCATAAGTTA--GCCATGCTGAGGTTGCAAAAGGG

Mouse GCCCTGAAGGCAATTTCTAAGTTGGATAGTTACAGACTGTTTATAATTGGACCTATTATTACCTTGCATAAGTTA--GCTATGTTGAGGTTGCAAAAGGA

110 120 130 140 150 160 170 180 190 200

....|....|....|....|....|....|....|....|....|....|....|....|....|....|....|....|....|....|....|....|

Human TAAGAAATTACCCTTAAGGGATGAAAATTCTTTAGAATAGAATGCAGTAGGCCCACGCAAACAACAACGTGTATAAATAGCTTAAATTAGCCTGTTTTTG

Chimp TAAGAAATTACCCTTAAGGGATGAAAATTCTTTGGAATAGAATGCAGTAGGCCCACGCAAACAACAACGTGTATAAATAGCTTAAATTAGCCTGTTTTTG

Orangutan TAAGAAATTACCCTTAAGGGATGAAAATTCTTTGGAATAGAATGCAGCAGGCCCACGCAAACAACAACGTGTATAAATAGCTTAAATTAGCCTGTTTTTG

Rhesus TACGAAATTACTCTTAAGGGATGAAAATTCTTTGGAATAGAATACAGTAGGCCCACACAAACAAGAATGTGTATAA--------AATTAGCCTGTTTTTG

Marmoset TAAGAAATTACCCTTAAGAGATGAAAATTCTTTGGAATAGAATGCAGTAG-CCCACACAAACAGGAATGTGCATAAATAGCTTAAATTAGCCTGTGTTTG

Cat TAGGAAATTATCCTAAAGGAACAAAATTGTTT-----------ACAGTTGGGCCATCTAAACAAGCATGTGTATAAATAGCTTGAATTAGCCTATGTTTG

Horse TAGGAAATTATCCTAAAGGAATGAAATTGTTT-----------GCAGTAGACCCATCTAAACAAGAATGTGTATAAATAGCTTGAATTAGCCTGTGTTTG

Rabbit TAGGGCGTTGTCATAAAGGGATTAAAACTCTTTGGAATAGAATGTAGTAGGCCTATCTAAGCAAGAGCTTGTACAGGTCGCTTACATTAGCCTGTGTTTG

Rattus CGAGAGGTTCCTC----AGGATGGAAATTGTTT----------GGAATAG---------GACAAGGGCATGCCCAAACCGCTTCAGTTA-TCTGTGTTTG

Mouse CGGGAGGTTACTC----AGGATGGAAACTGTTT----------GGAATAG---------AACAAGGACATGCCCAAACCGTTTCAATTA-TCTGTGTTTG

210 220 230 240 250 260 270

....|....|....|....|....|....|....|....|....|....|....|....|....|....|.

Human C-----CTAAGTACATTAACTCAAAAGGAAACTCGGTTTAAGAAGTTCACTCCAAAAATAGTGCAAGAAGC

Chimp C-----CTAAGTACATTAACTCAAAAGGAAACTTGGTTGAAGAAGTTCACTCCAAAAATAGTGCAAGAAGC

Orangutan C-----CTAAGTACATTAACTCAAAAGGAAACTTGGTTTAAGAAGTTCACTCTAAAAATAGCGCAAGAAGC

Rhesus C-----CTAAGTACATTAACTCAAAAGGAAACTTGGTTTAAGAAGTTCACTCTAAAAATAGTGCAAGAAGC

Marmoset C-----ATAAGGACATTAACTGGAAAGGAAACTTGGTTTAAGAAGTTCACTTTAAAAATTGTGCAAGAAGC

Cat CTTGTGTTAAGTACTTGAACTCAAAAGGAAACTTGGTTTAAAAAGGTCATTCTAAAAATAGTGCAAGTAGC

Horse CTTGTGCTAAGCACATTTACTCTAAAGGAACCTTCTTTTAAAAAATTAAATCTAGAAACAGTGCAAGTAGC

Rabbit CTTGTGCTAAGTATATTAACCCAGAAAGAAACTTGGTTTTTGAAGGTCAATCTAAAAACAGTTCTGGAAGC

Rattus CTTG--T-----GCCGTGATCCAGAAAGAGATTTCATGTAGGAAT-CAGTCTTAAAATAAAAGCTGGGTC-

Mouse TTTG--TTTTGTGTCATGATCCTGAAAGAAATTTTGTGTAGGAGTTCAATCTTAAAATGAGCGTTGAGTT-

**S5 Fig. Homology of the mouse *Ccnb1ip1* insulator DNA sequence with orthologous sequences in humans, chimps, orangutans, rhesus macaques, marmosets, cats, horses, rabbits, and rats.** Conserved sequences are highlighted.
