# Supplementary material for: An Insulator Element Located at the Cyclin B1 Interacting Protein 1 Gene Locus Is Highly Conserved among Mammalian Species
Source: PLoS One. 2015 Jun 25;10(6):e0131204. doi: 10.1371/journal.pone.0131204 (PMC4481373; doi:10.1371/journal.pone.0131204)
Supplement: S6 Fig — The position of the homologous sequence is indicated by a vertical bar. (DOCX) [file pone.0131204.s006.docx]

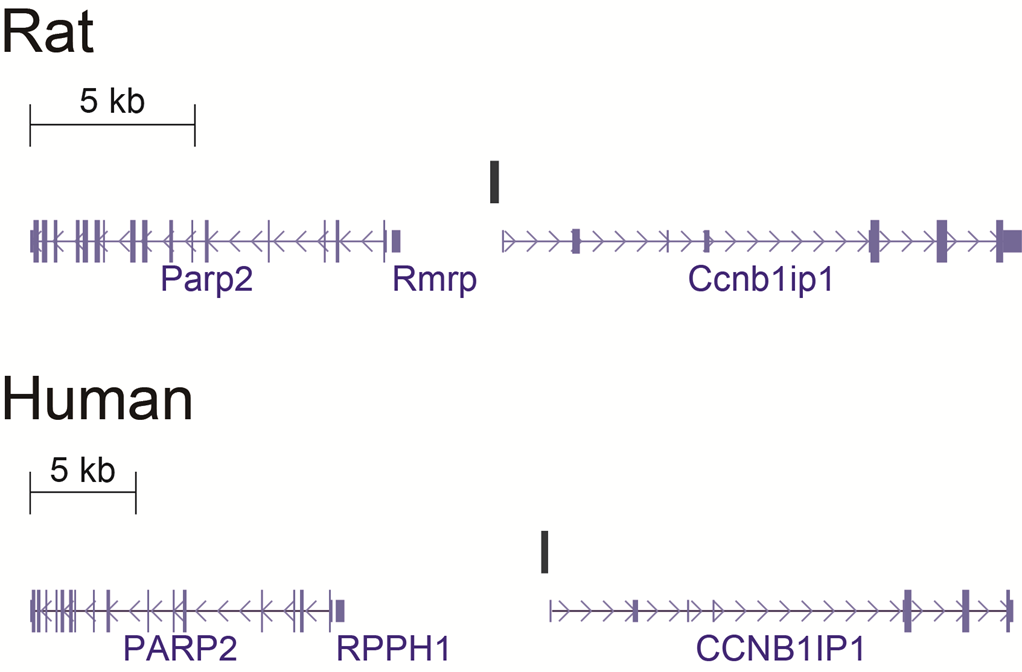


**S6 Fig. Location of the genomic region homologous to the mouse *Ccnb1ip1* insulator sequence at the *PARP2-CCNB1IP1* locus in the rat (upper) and human (lower) genomes.** The position of the homologous sequence is indicated by a vertical bar.
