# Supplementary material for: An Insulator Element Located at the Cyclin B1 Interacting Protein 1 Gene Locus Is Highly Conserved among Mammalian Species
Source: PLoS One. 2015 Jun 25;10(6):e0131204. doi: 10.1371/journal.pone.0131204 (PMC4481373; doi:10.1371/journal.pone.0131204)
Supplement: S7 Fig — Sequences for each exon were aligned using BioEdit (http://www.mbio.ncsu.edu/bioedit/bioedit.html). Conserved sequences are highlighted. (DOCX) [file pone.0131204.s007.docx]

**Exon1**

10 20 30 40 50

....|....|....|....|....|....|....|....|....|....|....|....

Human_Exon1 ACTTCCCAAGGCGACTTCCTGTCTCTCCACTTTCTTTCCCTCTCCGTTTTGGTGGGCTG

JF1_Exon1 --------------------------------------------------GATGGTCTG

Rat_Exon1 --------------------------------------------------GGTGGTCTG

**Exon2**

10 20 30 40 50 60 70 80 90 100

....|....|....|....|....|....|....|....|....|....|....|....|....|....|....|....|....|....|....|....|

Human_Exon2 ------GTTGAAGATGAAATCCACTGAGGAGGGAAGTCCAGCACCCTGTGTGCCAGTCCAG------------AACTGGCCCA--TCTGTAGACCCCCTG

JF1_Exon2 TTCCAGGTTGAAGATGAATTGTGTCCAGGAAAGAG--CTGGGACTGTAGCACTCAGCCCCG-------------CCCGGCATA------GAGGCCCTGTG

Rat_Exon2 TTTCAGGTTGAAGATGAAACGTATCCAGGAAAGAG--CTGGGACTGTAGCACTCAGCCCAGTGGCTGAGTCCAGCCCAGCACAGGCCCAGAGGCCCTGCA

110 120 130 140 150 160 170 180 190 200

....|....|....|....|....|....|....|....|....|....|....|....|....|....|....|....|....|....|....|....|

Human_Exon2 AAAATC-----ATATG---GGCTTGGATTTGGATATTCTCAAC--AGAAAGGGTTAAAGGCTGATGGTACCTAAAGCCTGGTACTTGAATTTTGATCAAG

JF1_Exon2 AAAACCCCAGGATATC--TAAAGTGGGCTTGGACATTCTCTCTCAAGGAAGGTTTAGAGGCTGATGGGAC-TATATATAAATGCAG--------------

Rat_Exon2 CAACCCCCAGAATATCTCTAAAGTGGGCTTGGATATTCTC-----AAGAAGGTTTAAAGGCTGATGGCAC-TGTA----AATGCAG--------------

210 220 230

....|....|....|....|....|....|

Human_Exon2 ATAAGCTGCCTTAAGTTCTCTTCATTACAC

JF1_Exon2 ------------------------------

Rat_Exon2 ------------------------------

**Exon3**

10 20 30 40 50 60 70

....|....|....|....|....|....|....|....|....|....|....|....|....|....|....|....

Human_Exon3 AAATGATCCTAGATAATTGATAGATCCTGTGGTTCAACT-GGATTTCTAGATAGAAGCTGGATTCATGTGATGCCAGAG

JF1_Exon3 --ATGATGGTAAGCAGTT----GCTCTGGTATTTCAATTTGGATTCCTAGATT-AAACTGGATTCATTTGATAA-----

Rat_Exon3 ATGAGATGGCAGCCCGTTT---GCTCTGGTATTTCCACTTGGATTCCTAGGTT-AAGCTGGATGCATTTGTTAACAG--

**Exon4**

10 20 30 40 50 60 70 80 90 100

....|....|....|....|....|....|....|....|....|....|....|....|....|....|....|....|....|....|....|....|

Human_Exon4 ---------------------------------GAGTAAAATTTCAAGAGACTGAAACCAGATCTGAGTT-TCGCTGTTCCAGTCTGGACCTCTTTGGTG

JF1_Exon4 CAGAAATGAAGGATGAAATACAAGAAGTACGAATATTGAACATTCAGGAGAGAACATCAGGTTGATAGCAATCCCATTTCCTGTCTGGACCATC---ATG

Rat_Exon4 ---AAATGAGGGGTAAAACCCAAGAAGTACAAATGTTCAACAGTCAGGAGAGAACACCAGGTTGACAGCA-TCACATTTCCTGTCTGGACCTTC---ATG

110 120 130 140 150

....|....|....|....|....|....|....|....|....|....|

Human_Exon4 CTGTAAATCC-TGGATATACTGTAGATGAGTACTGCGTTTTTCTTTTATG

JF1_Exon4 CTGTAAAACCATGGTTGTATGAAAAATGATAGTTGCATTGTTTAATG---

Rat_Exon4 CTATAAACC---AGTTGTATGAAAAA-AATGATCGCATGGTTTAATG---

**S7 Fig. Alignments of human, mouse (JF1), and rat sequences of exons 1 to 4 of the *Ccnb1ip1* gene.** Sequences for each exon were aligned using BioEdit (http://www.mbio.ncsu.edu/bioedit/bioedit.html). Conserved sequences are highlighted.
