# Supplementary material for: An Insulator Element Located at the Cyclin B1 Interacting Protein 1 Gene Locus Is Highly Conserved among Mammalian Species
Source: PLoS One. 2015 Jun 25;10(6):e0131204. doi: 10.1371/journal.pone.0131204 (PMC4481373; doi:10.1371/journal.pone.0131204)
Supplement: S8 Fig — CTCF ChIP-seq data from the ENCODE project (https://genome.ucsc.edu/ENCODE/) are shown. Red rectangles indicate the location of an insulator for each locus: 5’HS4 insulator at HBB locus, the putative CCNB1IP1-insulator, and the ANK1 insulator [18]. Whereas CTCF peaks were observed in the majority of the cell lines at the 5’HS4 insulator, no peaks were observed at CCNB1IP1 and ANK1 insulators. (PPTX) [file pone.0131204.s008.pptx]

## Slide 1
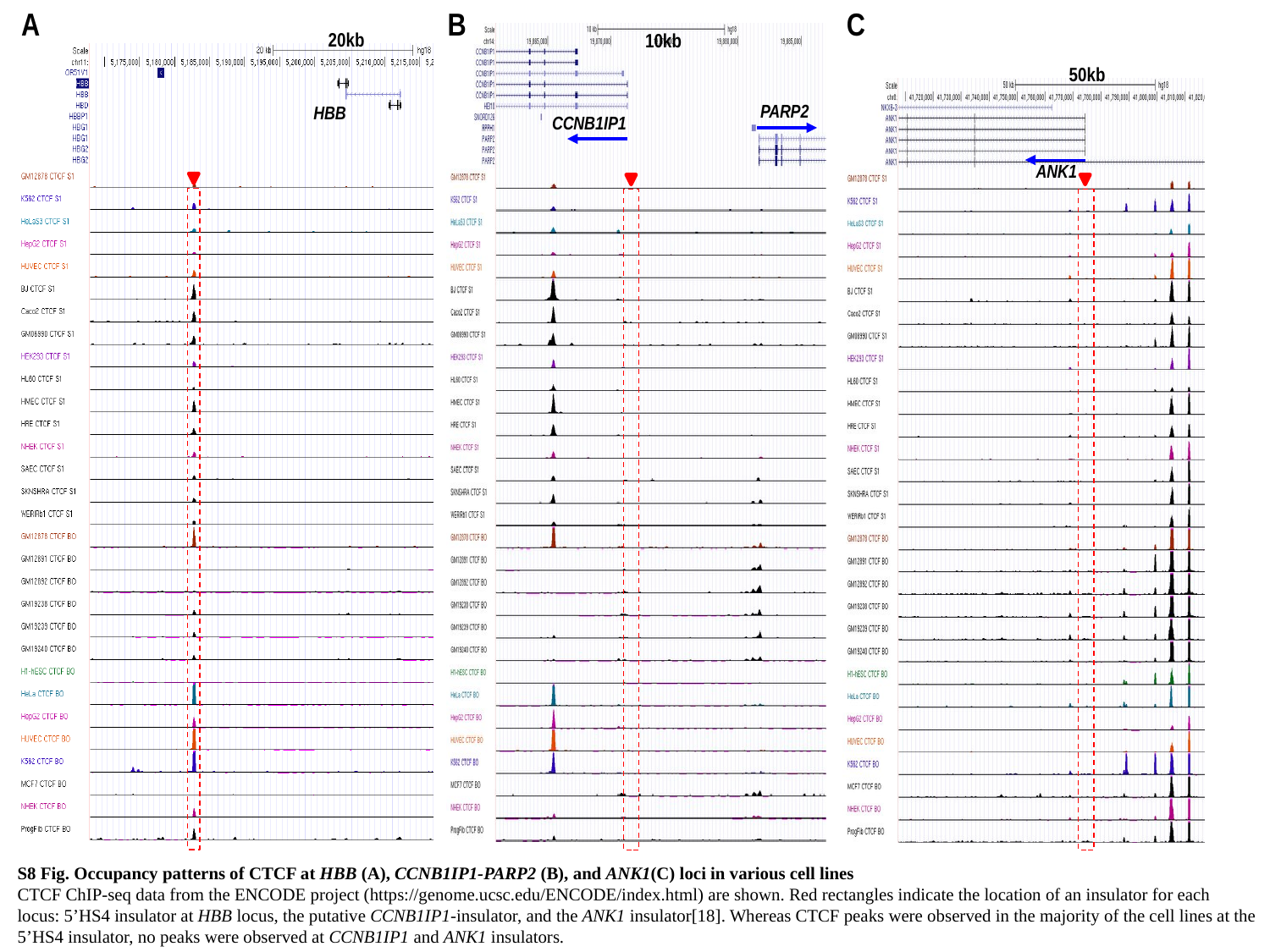

CTCF binding patterns at the human CCNB1IP1 locus
CTCF binding patterns at the human beta-globin locus
A
B
C
20kb
10kb
50kb
PARP2
HBB
CCNB1IP1
ANK1
S8 Fig. Occupancy patterns of CTCF at HBB (A), CCNB1IP1-PARP2 (B), and ANK1(C) loci in various cell lines
CTCF ChIP-seq data from the ENCODE project (https://genome.ucsc.edu/ENCODE/index.html) are shown. Red rectangles indicate the location of an insulator for each locus: 5’HS4 insulator at HBB locus, the putative CCNB1IP1-insulator, and the ANK1 insulator[18]. Whereas CTCF peaks were observed in the majority of the cell lines at the 5’HS4 insulator, no peaks were observed at CCNB1IP1 and ANK1 insulators.
