# Supplementary material for: An Insulator Element Located at the Cyclin B1 Interacting Protein 1 Gene Locus Is Highly Conserved among Mammalian Species
Source: PLoS One. 2015 Jun 25;10(6):e0131204. doi: 10.1371/journal.pone.0131204 (PMC4481373; doi:10.1371/journal.pone.0131204)
Supplement: S9 Fig — “Histone Modifications by ChIP-seq” and “Chromatin State Segmentation by HMM” tracks of ENCODE/Broad Institute data (http://genome.ucsc.edu/ENCODE/) are shown for GM12878, H1-hESC, K562, A549, HeLa-S3, HepG2, and HUVEC cells. (PPTX) [file pone.0131204.s009.pptx]

## Slide 1
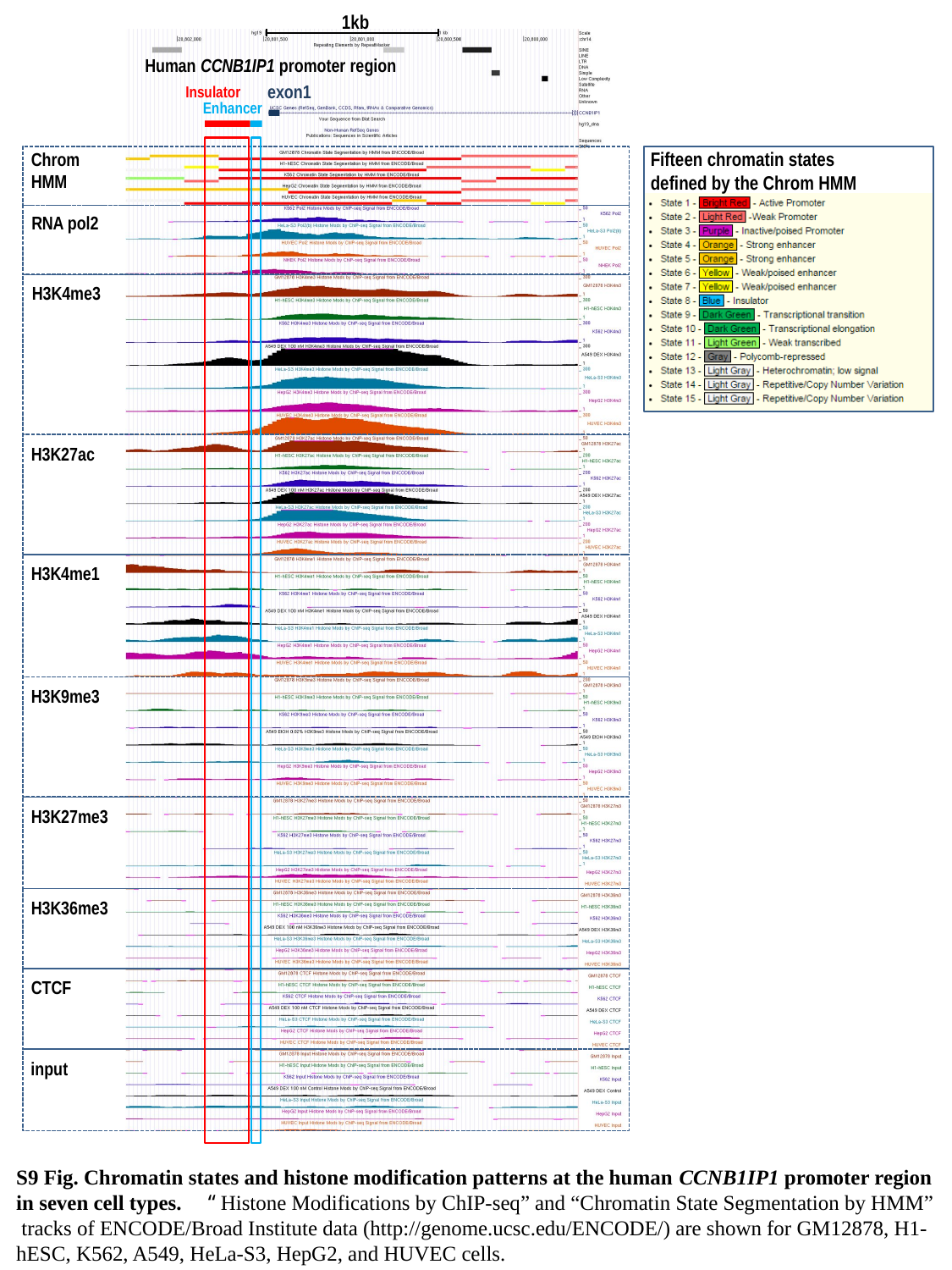

1kb
Human CCNB1IP1 promoter region
exon1
Insulator
Enhancer
Chrom
HMM
RNA pol2
H3K4me3
H3K27ac
H3K4me1
H3K9me3
H3K27me3
H3K36me3
CTCF
input
Fifteen chromatin states
defined by the Chrom HMM
S9 Fig. Chromatin states and histone modification patterns at the human CCNB1IP1 promoter region in seven cell types.　“Histone Modifications by ChIP-seq” and “Chromatin State Segmentation by HMM” tracks of ENCODE/Broad Institute data (http://genome.ucsc.edu/ENCODE/) are shown for GM12878, H1-hESC, K562, A549, HeLa-S3, HepG2, and HUVEC cells.
